# Correction: Lana et al. Sacral Bioneuromodulation: The Role of Bone Marrow Aspirate in Spinal Cord Injuries. *Bioengineering* 2024, *11*, 461

**DOI:** 10.3390/bioengineering11111166

**Published:** 2024-11-19

**Authors:** José Fábio Lana, Annu Navani, Madhan Jeyaraman, Napoliane Santos, Luyddy Pires, Gabriel Silva Santos, Izair Jefthé Rodrigues, Douglas Santos, Tomas Mosaner, Gabriel Azzini, Lucas Furtado da Fonseca, Alex Pontes de Macedo, Stephany Cares Huber, Daniel de Moraes Ferreira Jorge, Joseph Purita

**Affiliations:** 1 Department of Orthopedics, Brazilian Institute of Regenerative Medicine (BIRM), Indaiatuba 13334-170, SP, Brazil; 2 Regenerative Medicine, Orthoregen International Course, Indaiatuba 13334-170, SP, Brazil; 3 Clinical Research, Anna Vitória Lana Institute (IAVL), Indaiatuba 13334-170, SP, Brazil; 4 Medical School, Max Planck University Center (UniMAX), Indaiatuba 13343-060, SP, Brazil; 5 Comprehensive Spine & Sports Center, Campbell, CA 95008, USA; 6 Department of Orthopaedics, ACS Medical College and Hospital, Chennai 600077, Tamil Nadu, India; 7 Medical School, Federal University of São Paulo (UNIFESP), São Paulo 04024-002, SP, Brazil

## Missing Citation

In the original publication [1], “Flett, S.; Garcia, J.; Cowley, K.C. Spinal electrical stimulation to improve sympathetic autonomic functions needed for movement and exercise after spinal cord injury: A scoping clinical review. *J. Neurophysiol.* **2022**, *128*, 649–670” was not cited for Figure 2. The citation has now been inserted in the Figure 2 as below:

**Figure 2.** Medullary spinal control center for the integration of sympathetic autonomic support. Reproduced with permission from [136], Journal of Neurophysiology, granted copyright by Canadian Science Publishing, published by American Physiological Society, 2022.

The authors state that the scientific conclusions are unaffected. This correction was approved by the Academic Editor. 
